# Inhibition of human chondrosarcoma cell growth via apoptosis by peroxisome proliferator-activated receptor-γ

**DOI:** 10.1038/sj.bjc.6600241

**Published:** 2002-04-22

**Authors:** K Nishida, T Furumatsu, I Takada, A Kawai, A Yoshida, T Kunisada, H Inoue

**Affiliations:** Department of Orthopaedic Surgery, Faculty of Medicine, Okayama University Medical School, 2-5-1 Shikata-cho, Okayama City, Okayama 700-8558, Japan

**Keywords:** chondrosarcoma, apoptosis, PPARγ, 15d-PGJ_2_

## Abstract

A rare immunohistochemical study using 28 surgical sections of human chondrosarcoma revealed that 67.9% of tumour cells had weak (10-40%) or strong (>40%) positive immunoreaction for peroxisome proliferator-activated receptor-γ. The expression of peroxisome proliferator-activated receptor-γ mRNA and protein in human chondrosarcoma cell line OUMS-27 was also determined by reverse transcription-polymerase chain reaction and immunocytochemistry, respectively. Furthermore, the effects of peroxisome proliferator-activated receptor-γ ligands on cell proliferation and survival were investigated in OUMS-27 cells. Pioglitazone, a selective ligand for peroxisome proliferator-activated receptor-γ, and 15-deoxy-Δ^12,14^-prostaglandin J_2_ (15d-PGJ_2_), a putative endogenous ligand for peroxisome proliferator-activated receptor-γ, inhibited the proliferation of OUMS-27 cells in a dose-dependent manner. The mechanism of cytotoxic effects of 15d-PGJ_2_ was via apoptosis as shown by DNA fragmentation using TUNEL stain and DNA ladder formation, and by ultrastructural analysis using transmission electron microscopy. Flow-cytometric analysis using annexin-V-fluorescein and propidium iodide detected the early change of apoptosis, as well as necrosis of OUMS-27 cells at 4 h after co-incubation with 15d-PGJ_2_. These results suggest that peroxisome proliferator-activated receptor-γ may play a significant role in the pathogenesis of chondrosarcoma, and peroxisome proliferator-activated receptor-γ ligands, especially 15d-PGJ_2_, may be of therapeutic value in the treatment of human chondrosarcoma.

*British Journal of Cancer* (2002) **86**, 1303–1309. DOI: 10.1038/sj/bjc/6600241 www.bjcancer.com

© 2002 Cancer Research UK

## 

Peroxisome proliferator-activated receptors (PPARs) are members of the nuclear receptor superfamily of ligand-activated transcription factors. To date, three mammalian PPAR subtypes have been isolated, and termed PPARα, PPARδ and PPARγ, which heterodimerise with the retinoid X receptors (Kliewer *et al*, 1992). PPARα is predominantly expressed in the liver, and studies have focused on its role in lipid metabolism (Latruffe and Vamecq, 2000). PPARδ is ubiquitously expressed, but its function is not well understood. Although PPARγ was first found to be expressed at high levels in adipose tissue, and functions as a critical regulator of adipocyte differentiation and fat metabolism (Chawla *et al*, 1994), the expression of PPARγ is not restricted to the cells of the adipocytic lineage, and has been demonstrated in many other human cell types including chondrocytes (Bordji *et al*, 2000).

Induction of differentiation and apoptosis in cancer cells by ligands of PPARγ is a novel therapeutic approach against malignant tumours. Recent studies reported that 15-deoxy-Δ^12,14^-prostaglandin J_2_ (15d-PGJ_2_), the most potent endogenous ligand for PPARγ, and the synthetic PPARγ ligand thiazolidinedione (TZD) can induce terminal differentiation and growth inhibition of human liposarcoma (Tontonoz *et al*, 1997), breast (Elstner *et al*, 1998; Yee *et al*, 1999), prostate (Kubota *et al*, 1998), colon (Sarraf *et al*, 1998; Kitamura *et al*, 1999), gastric (Takahashi *et al*, 1999; Sato *et al*, 2000), lung (Chang and Szabo, 2000; Tsubouchi *et al*, 2000), and pancreas (Motomura *et al*, 2000; Elnemr *et al*, 2000) cancer cells, as well as hepatoma cells (Koga *et al*, 2001) *in vitro* and *in vivo*. The *in vivo* induction of histological and biochemical differentiation of liposarcomas by clinical trial of troglitazone administration was reported by Demetri *et al* (1999). Among these patients the immunohistochemical expression of Ki-67, a marker of cell proliferation was markedly reduced. The authors of a similar *in vivo* trial have suggested the clinical efficacy of troglitazone in prostate cancer (Mueller *et al*, 2000). Although the precise mechanism is still unknown, the interference in tumour cell growth by PPARγ ligands may have a favourable clinical effect.

Chondrosarcoma is a relatively rare malignant bone tumour accounting for only 3–6% of primary bone tumours (Mirra, 1989; Schajowics, 1994; Unni, 1996). The histologic grade of chondrosarcoma which indicates the differentiation status of tumour cells, is one of the most important factors for the prognosis of the disease (Evans *et al*, 1977). The surgical treatment of chondrosarcoma with adequate marginal resection is effective for low-grade chondrosarcoma with better clinical outcomes (Ozaki *et al*, 1996). However, for high-grade chondrosarcoma, the prognosis is poor even after adequate surgery (Sheth *et al*, 1996), the efficacy of systemic chemotherapy or irradiation is still unclear. Thus, the development of clinically useful agents which inhibit cell growth and induce cell death may be useful in the treatment of chondrosarcoma.

In the current study, the first of its kind, the authors examined the expression of PPARγ both in surgically resected sections of human chondrosarcoma and OUMS-27 cells (Kunisada *et al*, 1998), a novel cell line established from a grade III human chondrosarcoma of a 65-year-old male. The examination of the effects of PPARγ ligands on OUMS-27 cells demonstrated that PPARγ ligands inhibited cell proliferation in a dose-dependent manner, and induced apoptosis of OUMS-27. The findings of the current study might contribute to the development of novel therapies for human chondrosarcoma.

## MATERIALS AND METHODS

### Immunohistochemistry on patient specimens and semiquantitative analysis

Immunohistochemistry was performed as previously described (Nishida *et al*, 2001) on formalin-fixed, paraffin-embedded tissues of human chondrosarcoma surgically resected from 28 patients. Briefly, sections were treated by polyclonal anti-human PPARγ antibody (Affinity BioReagents, Inc, Golden, CO, USA) diluted into 10 μg ml^−1^ with PBS containing 0.1% NaN_3_ and 0.1% bovine serum albumin (British Biocell International Co, UK). After incubation for 30 min at room temperature with 7.5 μg ml^−1^ of biotinylated goat anti-rabbit IgG (Vector Laboratories Inc, CA, USA), tissues were then treated by ABC method for 30 min, visualised with diaminobenzidine (DAB) (Sigma) in H_2_O_2_ (0.01%) for 5 min, and counterstained with methyl-green. Negative control was obtained in the same fashion but excluding the primary antibody. They were then examined under a light microscope.

Each specimen showed pathologically different grades of chondrosarcoma; grade I (*n*=20), grade II (*n*=6) and grade III (*n*=2). For the semiquantitative analysis of PPARγ positive tumour cells in the human tissue, the amount of the staining was evaluated in each of the three grades of chondrosarcoma.

### Cells and cell culture

The human chondrosarcoma cell line OUMS-27 was a generous gift from Dr Masayosi Namba (Okayama University Medical School, Japan). OUMS-27 is a cell line established from grade III chondrosarcoma of a 65-year-old patient. The cells were cultured in Dulbecco's modified Eagle's medium (DMEM) containing 450 mg dl^−1^ glucose, supplemented with 10% foetal bovine serum (FBS) (Life Technologies, Rockville, MD, USA), 100 U ml^−1^ penicillin, and 100 μg ml^−1^ streptomycin (Life Technologies). Cells were incubated at 37°C in a humidified atmosphere of 5% CO_2_ in air. Cells were sub-cultured at a split ratio of 1 : 3 every 7–10 days with 0.1% trypsin (Life Technologies), 5 mM EDTA solution. The medium was regularly changed twice a week.

### RNA extraction and RT–PCR

Total cellular RNA was extracted from confluent cells using the RNA *plus kit* (Bioprobe System, Montreuil, France). For reverse transcriptase-polymerase chain reaction (RT–PCR), RNA samples (5 μg) were reverse-transcribed to cDNA using reverse transcriptase (ReverTra Ace, Toyobo Co. Ltd., Osaka, Japan) and subsequently amplified by PCR using as a sense primer, 5′-TCTCTCCGTAATGGAAGACC-3′ and an antisense primer, 5′-GCATTATGAGACATCCCCAC-3′ as previously reported (Takahashi *et al*, 1999). Human adult normal adipose total RNA was purchased from BioChain Institute, Inc, CA, USA (Catalogue Number 061005). Samples were reverse-transcribed to cDNA and used for the positive control of PPARγ mRNA expression. The reaction conditions were as follows: initial denaturation at 94°C for 2 min and 30 cycles of amplification (30 s at 94°C, 30 s at 58°C, and 40 s at 72°C) in an automated thermal cycler (Perkin Elmer Applied Biosystems, Foster, CA, USA), followed by a final extension step of 4 min at 72°C. PCR products were run on 1% agarose gels.

### Immunocytochemistry

The expression of PPARγ at the protein level was determined by immunocytochemistry. Briefly, cells were cultured at a density of 1×10^5^ cells per well in 6-well plastic culture dishes (Primaria, France), and fixed in 2% paraformaldehyde in 0.1 M phosphate buffered saline (PBS, pH 7.4) for 30 min at room temperature, washed in PBS. After permeabilisation in 0.1% Triton X-100 (Nacalai Tesque Inc., Kyoto, Japan) for 5 min at room temperature, non-specific staining was blocked by 10% normal goat serum (Vector Laboratories, Burlingame, CA, USA). Immunocytochemistry for PPARγ was performed in the same fashion as described above.

### Reagents for activation of PPARγ

Pioglitazone, a selective ligand for PPARγ, was kindly provided from Takeda Chemical Industries (Osaka, Japan). 15d-PGJ_2_, a potent natural ligand for PPARγ, was purchased (from Biomol Research Laboratories, Inc, MI, USA). These PPARγ ligands were dissolved in dimethyl sulphoxide (DMSO) with a final concentration of 0.1% DMSO in the culture medium.

### Cell proliferation and viability assay

Cells were seeded at 1×10^4^ cells per well in a 96-well culture plate or 8-well culture plate (Coster, Cambridge, MA, USA) in 100 μl 10% FCS-DMEM and incubated for 24 h at 27°C. For the activation of PPARγ, cells were treated with increasing concentrations of pioglitazone and 15d-PGJ_2_ for 24 h.

To know the effects of pioglitazone and 15d-PGJ_2_ on the proliferation grade of cell growth, an immunocytochemical study was performed in the same manner as described above on the cells cultured in 8-well culture plate, using monoclonal anti-Ki-67 antibody (Zymed Laboratories Inc, CA, USA) as a primary antibody and anti-mouse IgG (Vector Laboratories Inc, CA, USA) diluted to a final concentration of 10 μg ml^−1^ as a secondary antibody. PBS without primary antibody was used as the negative control. Semi-quantitative analysis on the Ki-67-immunostained cells were performed by counting the positive cell number per total cells in four fields at a magnification of ×200 under light microscopy.

Cell viability in a 96-well culture plate was evaluated using the colorimetric MTT assay (Chemicon International, Inc, Temecula, CA, USA) according to the manufacturer's instructions. Quantitation was then conducted using Model 550 microplate reader (BioRad, Hercules, CA, USA) at 595-655 nm. Results of optimal density units per 1×10^4^ adherent cells were expressed as % MTT.

### DNA fragmentation analysis

Cells were cultured for 24 h in the presence or absence of 15d-PGJ_2_ (10 μg ml^−1^) at a density of 1×10^5^ cells per well in 6-well plastic culture dishes. Cells were fixed in 2% paraformaldehyde in PBS (30 min at room temperature). Cells were then doubly stained by nick end-labelling method to detect the fragmented DNA, and Hoechst 33258 (Wako, Osaka, Japan) to observe the nuclear morphology of the cells. Briefly, cells were first incubated with TdT reaction solution (2 μg terminal deoxynucleotidyle transferase (TdT) and 5 μg FITC-conjugated deoxyuridine triphosphate (dUTP) in 1200 μl TdT buffer). The cells were then stained by Hoechst 33258 (10 μg ml^−1^) for 5 min at room temperature. After thorough washing in PBS, cells were observed by fluorescent microscopy.

The Apoptotic DNA Ladder Kit (Roche Diagnostics GmbH, Mannheim, Germany) was used for DNA ladder assay according to the manufacturer's instructions. Briefly, cells were cultured in serum-free DMEM at a density of 2.5×10^6^ cells in 75 mm^2^ tissue culture flask (Greiner, Frickenhawen, Germany), stimulated with 15d-PGJ_2_ (10 μg ml^−1^) for 24 h. Cells were homogenised in lysis buffer, and DNA was isolated. The samples were separated on a 2% agarose gel and stained with 10 μg ml^−1^ ethidium bromide.

### FACS analysis using annexin-V-FITC

The expression of phosphatidylserine on the outer leaflet of apoptotic cell membranes was examined by fluorescence-activated cell sorter (FACS) analysis. Annexin-V-Fluos Staining Kit (Roche Diagnostics GmbH, Mannheim, Germany) was used according to the manufacturer's instructions to stain the apoptotic cells and to differentiate them from necrotic cells. Briefly, cells were seeded at a density of 1×10^6^ cells on 25 cm^2^ tissue culture flask (Becton Dickinson, Planklin Lakes, NJ, USA) in serum-free DMEM were pretreated with 15d-PGJ_2_ (10 μg ml^−1^) for 4, 8 and 24 h, washed with PBS and centrifuged at 200 **g** for 5 min. Cell pellets were then re-suspended in 100 μl of 2% Annexin-V-fluorescein labelling reagent in Hepes buffer containing 2% propidium iodide (PI) and incubated for 15 min at 15 to 25°C. After washing extensively, stained cells were analysed using a FACScan (Becton Dickinson, San Jose, CA, USA) and 1000 events in the live gate were recorded (a 515 nm bandpass filter for fluorescein and a filter >600 nm for PI detection).

### Transmission electron microscopy

Ultrastructural appearances of apoptotic cells were confirmed by electron microscopic study. Cells were first incubated with or without 15d-PGJ_2_ (10 μg ml^−1^) for 8 h. Then cells were scraped, pelletised, rinsed with PBS, and then fixed in 1% glutaraldehyde in PBS (pH 7.4) overnight at 4°C. After rinsing in PBS three times, cell pellets were dehydrated by a graded series of ethanol, and finally embedded in hydrophilic resin (LR-White). Semi-thin sections were first stained by toluidine blue and examined by light microscopy. Adjacent ultra-thin sections were then contrasted with aquenous uranyle acetate and lead citrate, and examined by a transmission electron microscope (TEM) (type 7100, Hitachi, Tokyo).

### Statistical analysis

The results are expressed as mean±s.e.m. Statistical analysis was performed by one way analysis of variance and subsequent Fisher's LSD test. *P*<0.05 was considered statistically significant.

## RESULTS

### Expression of PPARγ in human chondrosarcoma and OUMS-27 cells

**Figure 1:**
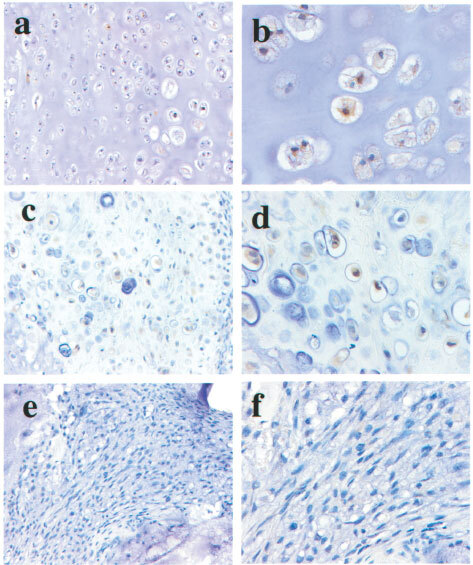
Expression of PPARγ protein of the cells in grade I (**A,B**) and grade II (**C,D**) areas within chondrosarcoma specimens from patients. Note there are few positive immuno-reactions in the cells in the grade III areas (**E,F**). Magnification; **A,C,E** 50×, **B,D,F** 100×.

Immunohistochemical examination revealed that human chondrosarcoma cells frequently express PPARγ at protein level (Figure 1

). In the nuclei of differentiated chondrocytes showed strong positive reaction for PPARγ, but not in the well differentiated hypertrophic chondrocytes nor undifferentiated mesenchymal cells. The results of the semiquantitative analysis for PPARγ-positive cells were summarised in Table 1

**Table 1:**
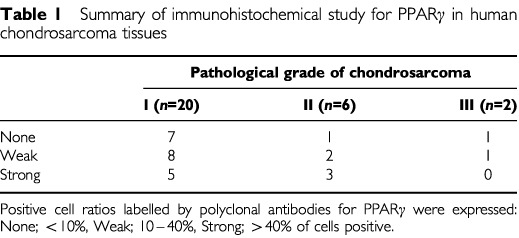
Summary of immunohistochemical study for PPARγ in human chondrosarcoma tissues

**Figure 2:**
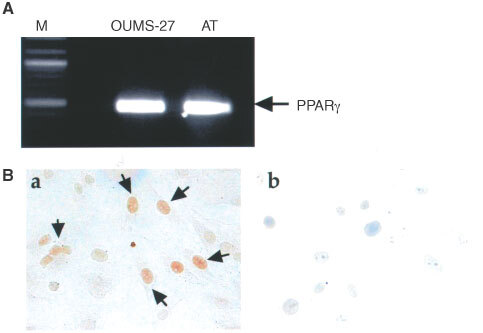
Expression of PPARγ in OUMS-27 cells. (**A**) Reverse transcription of total RNA into cDNA and RT–PCR was performed using specific primers for PPARγ. PPARγ was expressed at the mRNA level (474 bp) in OUMS-27 cells (arrow) as well as human adult normal adipose tissue. M: molecular control, AT: adipose tissue. (**B**) Immunocytochemical analysis of PPARγ in OUMS-27 cells was performed using polyclonal anti-PPARγ antibodies. a: Nuclear localisation of PPARγ protein (arrow heads), b: negative control.

. The positivity (the cutoff positivity of 10%) of chondrosarcoma cells were 65.0% in grade I, 83.3% in grade II, and 50.0% in grade III, and overall positivity was 67.9%. RT–PCR analysis was done to examine the expression of PPARγ mRNA in OUMS-27 cells. The predicted band size (474 bp) of PPARγ mRNA was clearly detected in OUMS-27 cells (Figure 2A

). Immunocytochemistry using polyclonal anti-human PPARγ antibody showed the nuclear localisation of PPARγ in OUMS-27 cells. Negative controls treated with non-immune serum or without primary antibodies showed no positive reaction (Figure 2B a,b). These data indicated that PPARγ is frequently expressed both in primary chondrosarcomas as well as cancer cell line OUMS-27.

### Inhibition of OUMS-27 cell proliferation by PPARγ ligands

**Figure 3:**
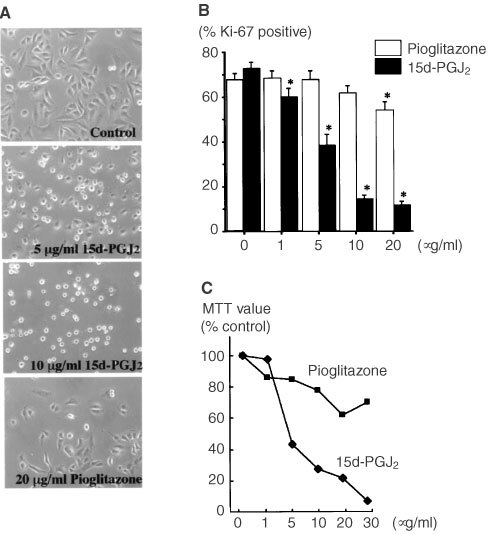
Effect of PPARγ ligands on OUMS-27 cell morphology and proliferation. OUMS-27 cells were cultured and treated with various concentrations of pioglitazone, 15d-PGJ_2_ or vehicle (0.1% DMS) for 24 h. (**A**) Under the phase contrast microscopy, cells showed relatively round shapes with loss of cell volume and detached from the dishes when treated with doses of 15d-PGJ_2_ 5 μg ml^−1^ or higher. High dose 15d-PGJ_2_ treatment (over 10 μg ml^−1^) caused cell detachment only 2 h after co-incubation. Pioglitazone treatment induced fewer morphological changes. (**B**) To identify the proliferative grade of cell growth, Ki-67-positive cell index was determined by counting the positive cells per total cells in four fields. Data are expressed as mean±s.e.m. of four fields. *P<0.01, when compared with vehicle only (ligands dose 0 μg ml^−1^). (**C**) Cell viability was determined by MTT assay. Although both PPARγ ligands inhibited cell growth in a dose dependent manner, the effects were more remarkable in 15d-PGJ_2_.

After the treatment with two different kinds of PPARγ ligands, the morphologic features of OUMS-27 cells were observed under phase contrast microscopy. Cells treated with vehicle only (0.1% DMSO) had a polygonal shape and adhered to the plastic surface. Cells treated with 15d-PGJ_2_ at 1 μg ml^−1^ appeared less active, but still had a polygonal appearance. When cells were treated with doses of 15d-PGJ_2_ at 5 μg ml^−1^ or higher, they showed relatively round shapes with cell shrinkage and apoptotic body-like structure and some cells could no longer adhere to the dish. The treatment with pioglitazone did not induce so much morphological change when compared with 15d-PGJ_2_ treatment (Figure 3A

). The results of immunostain for Ki-67 and MTT assay indicated that both pioglitazone and 15d-PGJ_2_ for 24 h inhibited the cell growth and reduced cell viability in a dose-dependent manner (Figure 3B,C). Interestingly, cell growth was markedly reduced when treated with 15d-PGJ_2_.

### Induction of apoptosis of OUMS-27 cells by PPARγ ligands

**Figure 4:**
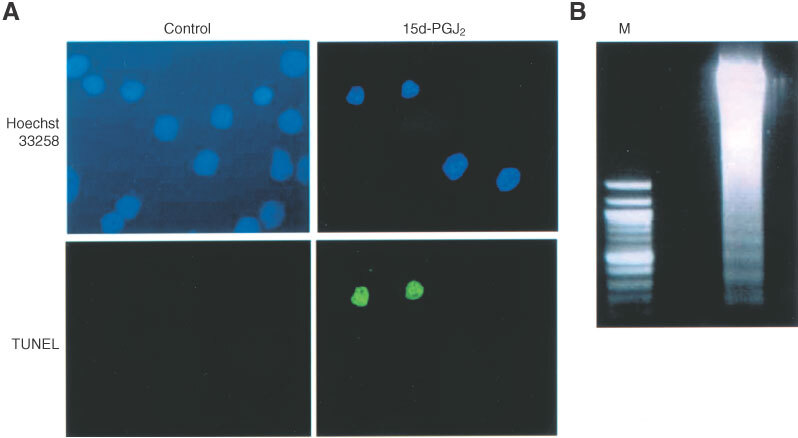
Induction of apoptosis by 15d-PGJ_2_ in OUMS-27. (**A**) OUMS-27 cells were incubated with or without 15d-PGJ_2_ (10 μg ml^−1^) for 24 h. Cells were doubly stained by Hoechst 33258 and simultaneously analysed by TUNEL stain, and observed by fluorescent microscopy. The cells with morphologically condensed nucleus were TUNEL positive, indicating the existence of fragmented DNA. (**B**) DNA was isolated TUNEL positive, indicating the existence of fragmented DNA. (**B**) DNA was isolated from the cells after co-incubation with 15d-PGJ_2_ (10 μg ml^−1^) for 24 h. A clear DNA-ladder formation was obtained after electrophoresis on a 2% agarose gel. M: marker.

**Figure 5:**
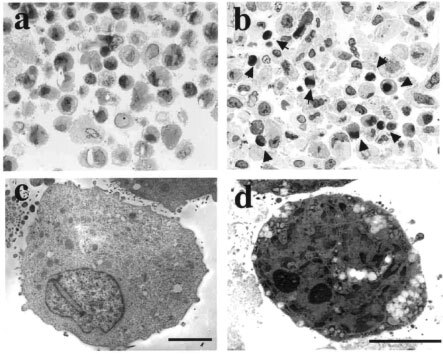
Cell morphology after incubation with or without 15d-PGJ_2_. The cells were treated by 10 μg ml^−2^ of 15d-PGJ_2_ for 8 h, and the cell pellet was embedded in hydrophilic resin. Semi-thin sections stained by toluidine blue show cell shrinkage and nuclear condensation (arrow heads) (**B**). TEM analysis of ultra-thin section after metal stain revealed typical apoptotic feature. Cells were small, and contained pyknotic nuclei, electron-dense granules and many vacuoles (**D**). (**A** and **C**) Control specimens without stimulation by 15d-PGJ_2_. Bar=5 μm.

**Figure 6:**
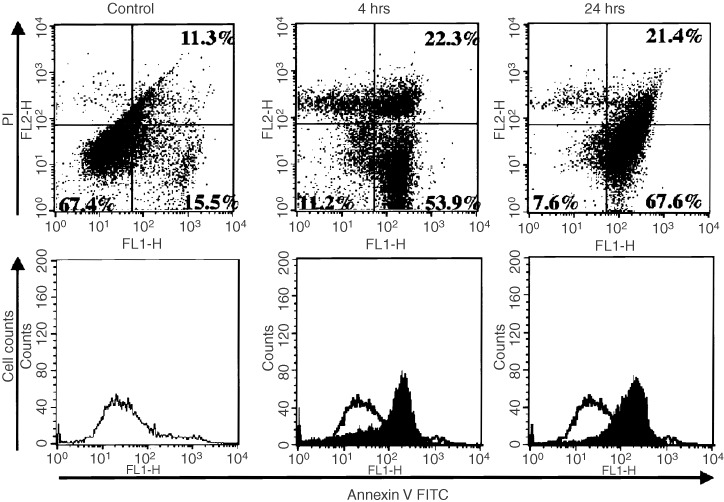
Effects of 15d-PGJ_2_ on OUMS-27 cells analysed by flow cytometry. The cells were incubated with or without 15d-PGJ_2_ (10 μg ml^−1^) for 4, 24 h and then analysed by flow cytometry. Cells were labelled with annexin-V-FITC and propidium iodide (PI) to distinguish apoptotic and/or necrotic cells from normal cells. Four hours incubation with 15d-PGJ_2_ caused early apoptotic change (annexin-V positive, PI negative) in 53.9% of the cells. The necrotic cells (annexin-V positive, PI positive) were also detected in 22.3%. The population of apoptotic cells increased to 67.6% at 24 h after treatment with 15d-PGJ_2_.

In order to confirm the apoptosis induced by PPARγ activation, DNA fragmentation was analysed both biochemically and morphologically. Hoechst staining showed condensed DNA chromatin morphology in OUMS-27 cells treated by 15d-PGJ_2_ for 24 h. The appearance of these cells was consistent with that of the cells positive for TUNEL staining which indicate DNA fragmentation (Figure 4A

). Treatment of OUMS-27 cells with 15d-PGJ_2_ (10 μg ml^−1^) for 24 h induced DNA ladder formation (Figure 4B). Semi-thin sections of LR-White embedded cells stained by toluidine blue showed a large number of OUMS-27 cells treated with 15d-PGJ_2_ with characteristic apoptotic features; cell shrinkage and nuclear condensation (Figure 5A,B

). TEM study revealed that untreated OUMS-27 cells have extensive rough-surfaced endoplasmic reticulum, thickened mitochondria and numerous cytoplasmic vesicles (Figure 5C). In contrast, when treated with 15d-PGJ_2_, the sections contained many apoptotic cells with condensed chromatin, many vacuoles in the cytoplasm and membrane budding (Figure 5D). FACS analysis indicated the population of apoptotic cells with PS at the outer membrane of the cells (annexin-V positive, PI negative) was approximately 53.9 and 67.6%, whereas those of necrotic cells (annexin-V positive, PI positive) were approximately 22.3 and 21.4% at 4 and 24 h, respectively, after treatment with 15d-PGJ_2_ (Figure 6

).

## DISCUSSION

There are few human chondrosarcoma cell lines with chondrocytic properties, OUMS-27 is a novel cell line established from grade III human chondrosarcoma by Kunisada *et al* (1998). OUMS-27 cells do not show contact inhibition after reaching confluence and grow rapidly in multiple layers. The cells express proteoglycan, as well as type I, II, III, IX and XI collagen after 120 passages, showing stable maintenance of the differentiated chondroctic properties. The transplantation of OUMS-27 cells into athymic mice resulted in formation of grade II (of III) chondrosarcoma at injected site (Kunisada *et al*, 1998). Thus, the OUMS-27 cell line appears to be a useful model for studies for chondrocyte function as well as the aetiology and treatment of human chondrosarcoma.

Recent studies strongly suggest the attractive concept that PPARγ ligands might be useful agents in the induction of cell differentiation or apoptosis in cancer cells. In the current study, the authors demonstrated for the first time that PPARγ is expressed in both surgically resected human chondrosarcoma and OUMS-27 cells. The frequent expression of PPARγ in chondrosarcoma cells indicated the involvement of PPARγ in the pathogenesis of chondrosarcoma and suggested the possible inhibitory effect on cancer cell growth and proliferation by PPARγ activation.

TUNEL staining, DNA ladder formation and TEM revealed that the type of cell death induced by 15d-PGJ_2_ was apoptosis. Interestingly, early apoptotic change, and the translocation of phosphatidylserine on the outer leaflet of the cell membrane were demonstrated only 4 h after co-incubation with 15d-PGJ_2_. To date, the exact intracellular signaling pathways leading to apoptotic cell death following specific activation of PPARγ is not fully understood, several factors have been suggested to be involved in PPARγ ligand-induced apoptosis. The activation and actin of caspases is believed to be a pivotal step in the mechanism of apoptosis. In JEG3 choriocarcinoma cells, 15d-PGJ_2_-induced apoptosis measured by a decline in MTT activity was significantly inhibited by the caspase inhibitor ZVAD-fmk, but the inhibitory effect was up to 50% (Keelan *et al*, 1999). Previous reports suggested the involvement of a caspase-3-independent mechanism in 15d-PGJ_2_-induced loss of MTT activity (Miyashita *et al*, 1998). We found that the inhibitors for caspase-1, 3, 8, 9, and ZVAD-fmk (2 μM) did not have significant effect on the 15d-PGJ_2_-induced loss of MTT activity in OUMS-27 (data not shown). It is speculated that the caspase-independent mechanism might be partly involved in the 15d-PGJ_2_-induced apoptosis in OUMS-27.

We also demonstrated that the ligands of PPARγ, both pioglitazone and 15d-PGJ_2_, inhibited cell growth and induced cell death in a dose dependent manner. The 15d-PGJ_2_ had more noticeable effects on OUMS-27 cell growth than pioglitazone. Thus, it is still unclear whether the effects of ligands on OUMS-27 cells were strictly due to PPARγ activation. The detailed analysis of the effects of ligands on the cells which do not express PPARγ should provide important clues to understand this phenomenon. Okura *et al* (2000) demonstrated that troglitazone can induce vascular smooth muscle cell apoptosis via the tumour suppressor p53, but not by PPARγ activation. We have previously shown by functional analysis of separated alleles in yeast (FASAY) assay that the p53-gene status of OUMS-27 is a mutant-type (Kunisada *et al*, 1998). The mutant p53 is known to inhibit the tumour-suppressor activity of wild-type p53 protein (Levine, 1997). In this line, mutant status of p53 might be involved in the pathway of the inhibition of OUMS-27 cell growth by PPARγ ligands. Other studies indicated that inhibition of transcriptional activation by nuclear receptors can be effected by competition for limited amounts of co-activators such as cAMP response element binding protein (CREB)-binding protein (CBP)/p300, Src-1 or TIF1 (Kamei *et al*, 1996; Göttlicher *et al*, 1998; Sheppard *et al*, 1998). It may be speculated that the difference in the recruitment of co-activators might influence the effectiveness of each ligand.

It has been shown that PPARγ has regulatory functions in the cell cycle by altering cell growth (Motomura *et al*, 2000; Okura *et al*, 2000; Morrison and Farmer, 1999). Recently, Motomura *et al* (2000) reported that activation of PPARγ by troglitazone inhibited cell growth and G1 arrest through the increase of cycline dependent kinase inhibitor p27^Kip1^ in human pancreatic carcinoma cells. They found that the effect of troglitazone on the proliferation of cancer cells was inhibited by antisense for p27^Kip1^. We have also found by immunohistochemistry that OUMS-27 cells express p27^Kip1^ at the protein level after incubation with 15d-PGJ_2_ (10 μg ml^−1^) for 4 h, but failed to show its expression by Western blot analysis (data not shown). Further cell cycle analysis on p27^Kip1^ is under investigation to elucidate the mechanism of PPARγ ligands-induced cell growth inhibition in OUMS-27 cells.

In conclusion, the current study showed that PPARγ activators induce apoptosis of human chondrosarcoma cells, suggesting that PPARγ activators might have potential therapeutic benefit in the treatment of chondrosarcoma. The signal transduction pathway for the induction of apoptosis is still unclear. Whether 15d-PGJ_2_ can also induce tumour cell death in experimentally transplanted chondrosarcoma models remains to be examined.

## References

[bib1] BordjiKGrillascaJPGouzeJNMagdalouJSchohnHKellerJMBianchiADaucaMNetterPTerlainB2000 Evidence for the presence of peroxisome proliferator-activated receptor (PPAR) alpha and gamma and retinoid Z receptor in cartilage. PPARγ activation modulates the effects of interleukin-1beta on rat chondrocytes J Biol Chem 275 1224312250 10766862 10.1074/jbc.275.16.12243

[bib2] ChangTHSzaboE2000 Induction of differentiation and apoptosis by ligands of peroxisome proliferator-activated receptor gamma in non-small cell lung cancer Cancer Res 60 11291138 10706135

[bib3] ChawlaASchwarzEJDimaculanganDDLazarMA1994 Peroxisome proliferator activated receptor (PPAR) gamma: adipose-predominant expression and induction early in adipocyte differentiation Endocrinology 135 798800 8033830 10.1210/endo.135.2.8033830

[bib4] DemetriGDFletcherCDMuellerESarrafPNaujoksRCampbellNSpiegelmanBMSingerS1999 Induction of solid tumor differentiation by the peroxisome proliferator-activated receptor-gamma ligand troglitazone in patients with liposarcoma Proc Natl Acad Sci USA 96 39513956 10097144 10.1073/pnas.96.7.3951 PMC22401

[bib5] ElnemrAOhtaTIwataKNinomiaIFushidaSNishimuraGKitagawaHKayaharaMYamamotoMTeradaTMiwaK2000 PPARγ ligand (thiazolidinedione) induces growth arrest and differentiation markers of human pancreatic cancer cells Int J Oncol 17 11571164 11078801 10.3892/ijo.17.6.1157

[bib6] ElstnerEMullerCKoshizukaKWilliamsonEAParkDAsouHShintakuPSaidJWHeberDKoefflerHP1998 Ligands for peroxisome proliferator-activated receptor-γ and retinoic acid receptor inhibit growth and induce apoptosis of human breast cancer cells in vitro and in BNX mice Proc Natl Acad Sci USA 95 88068811 9671760 10.1073/pnas.95.15.8806 PMC21158

[bib7] EvansHLAyalaAGRomsdahlMM1977 Prognostic factors in chondrosarcoma of bone: a clinicopathologic analysis with emphasis on histologic grading Cancer 40 818831 890662 10.1002/1097-0142(197708)40:2<818::aid-cncr2820400234>3.0.co;2-b

[bib8] GöttlicherMHeckSHerrlichP1998 Transcriptional cross-talk, the second mode of steroid hormone receptor action J Mol Med 76 480489 9660166 10.1007/s001090050242

[bib9] KameiYXuLHeinzelTTorchiaJKurokawaRGlossBLinSCHeymanRARoseDWGlassCKRosenfeldMG1996 A CBP integrator complex mediates transcriptional activation and AP-1 inhibition by nuclear receptors Cell 85 403414 8616895 10.1016/s0092-8674(00)81118-6

[bib10] KeelanJASatoTAMarvinKWLanderJGilmourRSMitchellMD1999 15-Deoxy-Delta(12,14)-prostaglandin J(2), a ligand for peroxisome proliferator-activated receptor-gamma, induces apoptosis in JEG3 choriocarcinoma cells Biochem Biophys Res Commun 262 57958510.1006/bbrc.1999.1257 10471366 10.1006/bbrc.1999.1257

[bib11] KitamuraSMiyazakiYShinomuraYKondoSKanayamaSMatsuzawaY1999 Peroxisome proliferator-activated receptor gamma induces growth arrest and differentiation markers of human colon cancer cells Jpn J Cancer Res 90 7580 10076568 10.1111/j.1349-7006.1999.tb00668.x PMC5925976

[bib12] KliewerSAUmesonoKNoonanDHeymanRAEvansRM1992 Convergence of 9-cis retinoic acid and peroxisome proliferator signalling pathways through heterodimer formation of their receptors Nature 358 771774 1324435 10.1038/358771a0 PMC6159883

[bib13] KogaHSakisakaSHaradaMTakagiTHanadaSTaniguchiEKawaguchiTSasatomiKKimuraRHashimotoOUenoTYanoHKojiroMSataM2001 Involvement of p21(WAF1/Cip1), p27(Kip1), and p18(INK4c) in troglitazone-induced cell-cycle arrest in human hepatoma cell lines Hepatology 33 1087109710.1053/jhep.2001.4024 11343236 10.1053/jhep.2001.24024

[bib14] KubotaTKoshizukaKWilliamsonEAAsouHSaidJWHoldenSMiyoshiIKoefflerHP1998 Ligand for peroxisome proliferator-activated receptor gamma (troglitazone) has potent antitumor effect against human prostate cancer both *in vitro* and *in vivo* Cancer Res 58 33443352 9699665

[bib15] KunisadaTMiyazakiMMiharaKGaoCKawaiAInoueHNambaM1998 A new human chondrosarcoma cell line (OUMS-27) that maintains chondrocytic differentiation Int J Cancer 77 854859 9714054 10.1002/(sici)1097-0215(19980911)77:6<854::aid-ijc10>3.0.co;2-1

[bib16] LatruffeNVamecqJ2000 Evolutionary aspects of peroxisomes as cell organelles, and of genes encoding peroxisomal proteins Biol Cell 92 389395 11132700 10.1016/s0248-4900(00)01083-2

[bib17] LevineAJ1997 p53, the cellular gatekeeper for growth and division Cell 88 323331 9039259 10.1016/s0092-8674(00)81871-1

[bib18] MiyashitaTNagaoKKrajewskiSSalvesenGSReedJCInoueTYamadaM1998 Investigation of glucocorticoid-induced apoptotic pathway: processing of caspase-6 but not caspase-3 Cell Death Differ 5 10341041 9894610 10.1038/sj.cdd.4400442

[bib19] MirraJ1989 Intramedullary cartilage- and chondroid-producing tumorsInBone Tumorspp439690Philadelphia: Lea and Febiger

[bib20] MorrisonRFFarmerSR1999 Role of PPARgamma in regulating a cascade expression of cyclin-dependent kinase inhibitors, p18(INK4c) and p21(Waf1/Cip1), during adipogenesis J Biol Chem 274 1708817097 10358062 10.1074/jbc.274.24.17088

[bib21] MotomuraWOkumuraTTakahashiNObaraTKohgoY2000 Activation of peroxisome proliferator-activated receptor gamma by troglitazone inhibits cell growth through the increase of p27^KiP1^ in human pancreatic carcinoma cells Cancer Res 60 55585564 11034103

[bib22] MuellerESmithMSarrafPKrollTAiyerAKaufmanDSOhWDemetriGFiggWDZhouXPEngCSpiegelmanBMKantoffPW2000 Effects of ligand activation of peroxisome proliferator-activated receptor gamma in human prostate cancer Proc Natl Acad Sci USA 97 1099010995 10984506 10.1073/pnas.180329197 PMC27136

[bib23] NishidaKDoiTMatsuoMIshiwariYTsujigiwaHYoshidaAShibaharaMInoueH2001 Involvement of nitric oxide in chondrocyte cell death in chondro-osteophyte formation Osteoarthritis Cartilage 9 23223710.1053/joca.2000.0380 11300746 10.1053/joca.2000.0380

[bib24] OkuraTNakamuraMTakataYWatanabeSKitamiYHiwadaK2000 Troglitazone induces apoptosis via the p53 and Gadd45 pathway in vascular smooth muscle cells Eur J Pharmacol 407 227235 11068018 10.1016/s0014-2999(00)00758-5

[bib25] OzakiTLindnerNHillmannATodlRBlasiusSWinkelmannW1996 Influence of intralesional surgery on treatment outcome of chondrosarcoma Cancer 77 12921297 8608505 10.1002/(SICI)1097-0142(19960401)77:7<1292::AID-CNCR10>3.0.CO;2-X

[bib26] SarrafPMuellerEJonesDKingFJDeAngeloDPartridgeJBHoldenSAChenLBSingerSFletcherCSpiegelmanBM1998 Differentiation and reversal of malignant changes in colon cancer through PPARgamma Nat Med 4 10461052 9734398 10.1038/2030

[bib27] SatoHIshiharaSKawashimaKMoriyamaNSuetsuguHKazumoriHOkuyamaTRumiMAFukudaRNagasueNKinoshitaY2000 Expression of peroxisome proliferator-activated receptor (PPAR)gamma in gastric cancer and inhibitory effects of PPARgamma agonists Br J Cancer 83 1394140010.1054/bjoc.2000.1457 11044367 10.1054/bjoc.2000.1457 PMC2408786

[bib28] SchajowicsF1994 ChondrosarcomaInTumors and tumor like lesions of bonepp201230Berlin: Springer

[bib29] ShethDSYaskoAWJohnsonMEAyalaAGMurrayJARomsdahlMM1996 Chondrosarcoma of the pelvis. Prognostic factors for 67 patients treated with definitive surgery Cancer 78 745750 8756367 10.1002/(SICI)1097-0142(19960815)78:4<745::AID-CNCR9>3.0.CO;2-D

[bib30] SheppardKAPhelpsKMWilliamsAJThanosDGlassCKRosenfeldMGGerritsenMECollinsT1998 Nuclear integration of glucocorticoid receptor and nuclear factor-κB signaling by CREB-binding protein and steroid receptor coactivator-1 J Biol Chem 273 2929129294 9792627 10.1074/jbc.273.45.29291

[bib31] TakahashiNOkumuraTMotomuraWFujimotoYKawabataIKohgoY1999 Activation of PPARgamma inhibits cell growth and induces apoptosis in human gastric cancer cells FEBS Lett 455 135139 10428487 10.1016/s0014-5793(99)00871-6

[bib32] TontonozPSingerSFormanBMSarrafPFletcherJAFletcherCDBrunRPMuellerEAltiokSOppenheimHEvansRMSpiegelmanBM1997 Terminal differentiation of human liposarcoma cells induced by ligands for peroxisome proliferator-activated receptor gamma and the retinoid X receptor Proc Natl Acad Sci USA 94 237241 8990192 10.1073/pnas.94.1.237 PMC19300

[bib33] TsubouchiYSanoHKawahitoYMukaiSYamadaRKohnoMInoueKHlaTKondoM2000 Inhibition of human lung cancer cell growth by the peroxisome proliferator-activated receptor-gamma agonists through induction of apoptosis Biochem Biophys Res Commun 270 40040510.1006/bbrc.2000.2436 10753637 10.1006/bbrc.2000.2436

[bib34] UnniKK1996 Chondrosarcoma (primary, secondary, de-differentiated, and clear-cell)InDahlin' bone tumorspp71108Philadelphia: Lippincott-Raven

[bib35] YeeLDSabourinCLLiuLLiHMSmithPJSeewaldtVKnissDA1999 Peroxisome proliferator-activated receptor gamma activation in human breast cancer Int J Oncol 15 967973 10536181 10.3892/ijo.15.5.967

